# Abnormalities of Cerebral White Matter Microstructure in Children With New-Onset, Untreated Idiopathic-Generalized Epilepsy

**DOI:** 10.3389/fneur.2021.744723

**Published:** 2021-11-30

**Authors:** Ran Long, Yuting Wang, Lizhou Chen, Dingmei Deng, Lan Mei, Jingping Mou, Guangcai Tang, Fugang Han, Graham John Kemp, Qiyong Gong, Lihua Qiu

**Affiliations:** ^1^Department of Radiology, The Affiliated Hospital of Southwest Medical University, Luzhou, China; ^2^Department of Radiology, The Second People's Hospital of Yibin, Yibin, China; ^3^Huaxi Magnetic Resonance Research Center, West China Hospital of Sichuan University, Chengdu, China; ^4^Department of Musculoskeletal Biology, Institute of Ageing and Chronic Disease, University of Liverpool, Liverpool, United Kingdom

**Keywords:** epilepsy, white matter, diffusion, anisotropy, cognition

## Abstract

Despite evidence for microstructural brain alterations in epilepsy patients, little is known about how these develop with age and the progress of the disease. The aim of this study was to investigate microstructural abnormalities of the white matter (WM) in children with new-onset, untreated idiopathic-generalized epilepsy (IGE) using the MRI technique of diffusion tensor imaging (DTI). The study was approved by the institutional review board, and all individuals or their parents gave signed informed consent. In total, 45 patients with IGE (age 5–18 years, male: female 26:19) and 32 healthy controls (HCs; age 5–18 years, male: female 21:11) were included. Voxel-based analysis (VBA) was used to compare patients and controls, and Pearson correlation analysis was used to investigate relationships between altered DTI metrics and clinical parameters. Compared with controls, patients with IGE showed increased mean diffusivity (MD) in the left splenium of the corpus callosum, increased fractional anisotropy (FA) in the right WM of the superior and middle frontal gyri, increased axial diffusivity (AD) in the WM of right corona radiata and left occipital lobe, and decreased AD in the WM of the left thalamus and the right middle cerebellar peduncle. There was no correlation between the altered diffusion parameters and clinical measures. Our study demonstrated several distinct microstructural impairments in children with new-onset, untreated IGE, of which altered AD might be the most sensitive marker of dysmyelination. The increased FA in the IGE group might suggest an initiating or compensatory mechanism that is activated prior to cognitive decline in these children.

## Introduction

Epilepsy is one of the commonest non-communicable neurologic conditions and a significant cause of disability and mortality, affecting 70 million people worldwide (1). Idiopathic-generalized epilepsies (IGE) are characterized by absence, myoclonic, and generalized tonic-clonic seizures (GTCS), which can present alone or in various combinations. IGE mostly occurs in adolescence or adulthood and may result in significant morbidity and even death (2–4). According to the latest International League Against Epilepsy (ILAE) classification (5, 6), IGE includes 4-well established epilepsy syndromes—childhood absence epilepsy (CAE), juvenile myoclonic epilepsy (JME), juvenile absence epilepsy (JAE), and GTCS—although not all patients are classifiable in these terms.

MRI has been widely used to investigate *in vivo* structural and functional changes in neuropsychiatric diseases, such as epilepsy. The MRI technique of diffusion tensor imaging (DTI), which is sensitive to the directionally constrained water diffusion along myelinated axons (7), has provided new opportunities for studying hemispheric differences in microscopic fiber characteristics. Several parameters can be used to quantify this tissue water diffusion: fractional anisotropy (FA) and mean diffusivity (MD) are widely used as markers of the microstructural integrity of white matter (WM) (8, 9); radial diffusivity (RD) and axial diffusivity (AD) represent water diffusion perpendicular and parallel to the axonal fibers, respectively. The magnitude and direction of diffusivity depend on the WM microstructure (10) and can reveal epileptogenic lesions that are not visible using conventional MRI.

A previous study (11) using DTI tractography demonstrated that diffusivity variables reliably reflect the integrity of WM microstructures in patients with pediatric epilepsy. Abnormalities in WM microstructural integrity have been widely reported in focal epilepsy and IGE. However, most studies have focused on chronic epilepsy, and the results may have been influenced by both neurodevelopmental and disease processes.

Only three studies (12–14) have investigated WM abnormalities in new-onset epilepsy, two (12, 14) using region of interest (ROI) analysis methods and one (13) using voxel-based analysis (VBA). An ROI-based study of children with idiopathic epilepsy (12) found decreased FA in the posterior corpus callosum and cingulum and increased MD in the posterior corpus callosum. An ROI study in children with JME (14) found decreased FA in the dorsolateral prefrontal cortex, supplementary motor area, right thalamus, posterior cingulate, anterior corpus callosum, corona radiata, and middle frontal WM. A whole-brain analysis in children with generalized epilepsy (13) found no abnormalities in FA or the apparent diffusion coefficient (ADC, which is similar to MD), but found the decreased AD in the left middle temporal gyrus and the anterior cingulum and increased AD in the cerebellar WM. These divergent results in children with new-onset epilepsy maybe related to differences in the data analysis methods, sample sizes, participant demographics (seizure type, epilepsy duration, frequency of seizures, age at onset, and duration of anticonvulsant therapy), and scanners.

Because the seizure type, epilepsy duration, and antiepileptic drug may influence WM integrity, we wished to elucidate the earliest brain changes in the natural course of childhood epilepsy. We, therefore, performed DTI in children with new-onset, untreated IGE: our first aim was to establish whether this show altered WM integrity relative to age-matched controls. Because neuropsychological impairments have been reported in both chronic epilepsy (15, 16) and new-onset epilepsy (17), our second aim was to establish whether the changes in diffusion parameters correlate with clinical parameters, such as the age of onset, duration of epilepsy, and cognitive measures, assessed by the mini-mental state examination (MMSE).

## Materials and Methods

### Participants

The study was approved by the local Research Ethics Committee and institutional review board. Written informed content and developmental and clinical history pertinent to epilepsy and treatment of a child were obtained from the patient or the parent. The ILAE diagnostic criteria were used to diagnose epileptic seizures (18). The included patients all met the following criteria: (1) presence of typical clinical symptoms of IGE, such as loss of consciousness and no partial seizures; (2) interictal electroencephalography (EEG) showing 2–4 or 4–6 Hz generalized spike-waves (SW) or polyspike-waves (PSW) on normal background activity, or the presence of at least one typical attack recorded during video-EEG monitoring with epileptiform activity and negative provocation test results; (3) no focal abnormality on routine structural MRI examination; (4) no other developmental disabilities or neurological disorders; and (5) epilepsy diagnosed within the past 6 months and never treated with an antiepileptic drug. The healthy controls (HCs) were recruited through poster advertisements, and all met the following criteria: no history of (1) any initial precipitating event (e.g., simple or complex febrile seizures); (2) any seizure or seizure-like episode; (3) neurological, psychological, developmental, or systemic disease; or (4) loss of consciousness >5 min. All children were attending regular school and were right-handed. A total of 60 patients with IGE and 37 matched HCs were initially recruited from the Affiliated Hospital of Southwest Medical University. MMSE measurements and MRI scans were completed on the same day. Six patients and two controls were excluded because of failure to finish either the MRI scanning or the MMSE assessment. Nine patients and three controls with head motion artifacts were also excluded. Finally, 45 patients with IGE (such as 2 with CAE, 1 with JAE, 11 with JME, 21 with GTCS, and 10 with an unidentifiable subsyndrome) and 32 matched HCs were enrolled in the study.

### Data Acquisition

All participants were scanned using a single-shot spin-echo echo-planar imaging (EPI) sequence on a 3.0T MR scanner (Intera Achieva; Philips Medical Systems, Amsterdam, the Netherlands). The EPI sequence was performed in 40 non-collinear gradient directions with a *b*-value of 1,000 s/mm^2^, and a non-diffusion-weighted image was used to obtain diffusion-weighted images. The other parameters were as follows: repetition time (TR) 8,700 ms, optimized echo time (TE) 90–110 ms, the number of excitations (NEX) 1, field of view (FOV) 24 × 24 cm^2^, matrix size 128 × 128, in-plane resolution 1.875 mm, and slice thickness 3.5 mm. The average scanning time was 7 min. An average of 40 oblique axial slices was collected to cover the whole brain.

### Data Analysis

Preprocessing was carried out using the FSL 4.1 software package (Functional Magnetic Resonance Imaging of the Brain Software Library; http://www.fmrib.ox.au.uk/fsl). First, the data were corrected for head motion and eddy currents using affine registration to the b0 image volume. Next, brain extraction was performed to remove non-brain tissue from the whole-head image. Finally, parameter maps for FA and MD were calculated using dtifit, which fits a diffusion tensor model to each voxel and estimates the principal directions of diffusion. Also calculated were AD, which represents the highest diffusion in the voxel and is assumed to run parallel to the main axon fiber, and RD, which represents diffusion perpendicular to the main diffusion direction of the axon fibers. A VBA was then performed with SPM8 (Statistical Parametric Mapping; Welcome Trust Centre for Neuroimaging, London, England, http://fil.ion.ucl.ac.uk) running in MATLAB 2012b (MathWorks, Natick, MA, USA). Each b0 image was non-linearly normalized using the EPI template supplied with SPM8 to estimate the normalization parameter, which was applied to all parameter maps with each voxel sized 2 mm × 2 mm × 2 mm. Finally, the normalized parameter maps were smoothed using an isotropic Gaussian filter (6-mm full width at half maximum).

Voxel-wise comparisons of FA, MD, AD, and RD between patients with IGE and controls were performed using two-sample *t*-tests and a WM mask in SPM8 with age and sex as covariates. The statistical significance threshold was set to *P* < 0.005 at the voxel level on uncorrected t maps. The correction for multiple comparisons was performed using a Monte Carlo simulation [5,000 simulations, full width at half maximum (FWHM) = 8 mm, and cluster connection radius = 5 mm] with an AlphaSim corrected (http://www.restfmri.net/forum/) *P* < 0.05 at the cluster level. The regions that showed group differences in FA MD, AD, and RD were extracted for each patient, and partial correlations using age and sex as covariates were computed to examine the relationships between the altered DTI parameters and clinical measures (such as the age of onset, duration of epilepsy, and MMSE score).

## Results

### Clinical Features

Anatomical MRI data from 77 participants were included in the study: 45 patients with IGE and 32 HCs. The demographic and clinical characteristics of the subjects are given in Table 1. The baseline features of sex and age between the two groups (all *P* > 0.05) were generally well-balanced. There was no significant difference in MMSE scores between IGE and HC groups.

**Table 1:** Demographic and clinical information on epilepsy patients and healthy controls.

	**IGE** **(***n*** = 45)**	**HC** **(***n*** = 32)**	***P*** **-value**
Age (years)	10.6 (3.6)	10.6 (3.3)	0.95
Gender (male/female)	26/19	21/11	0.49
MMSE	23.8 (6.2)	26.2 (5.2)	0.09
Age of onset	10.6 (3.2)		
Duration of epilepsy (months)	3.4 (3.8)		

*IGE, idiopathic-generalized epilepsy patients; HC, healthy controls; MMSE, mini-mental state examination; apart from gender, values shown are means, with SD in parentheses*.

### MRI Results

**Figure 1:**
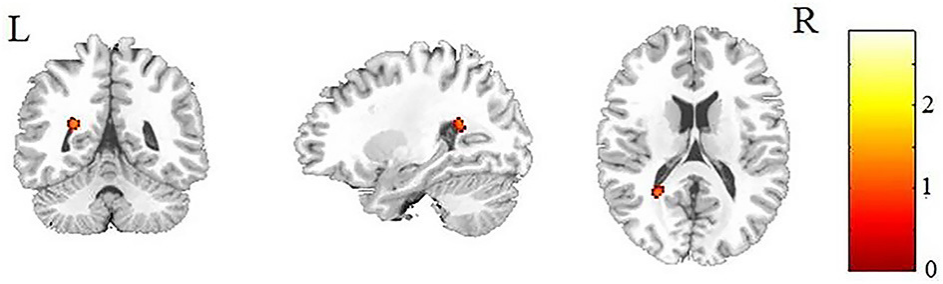
Patients with IGE showed increased MD (refer to color scale) in the WM of the left splenium of the corpus callosum compared with healthy controls after AlphaSim correction (*P* < 0.05). MD, mean diffusivity; WM, white matter.

**Figure 2:**
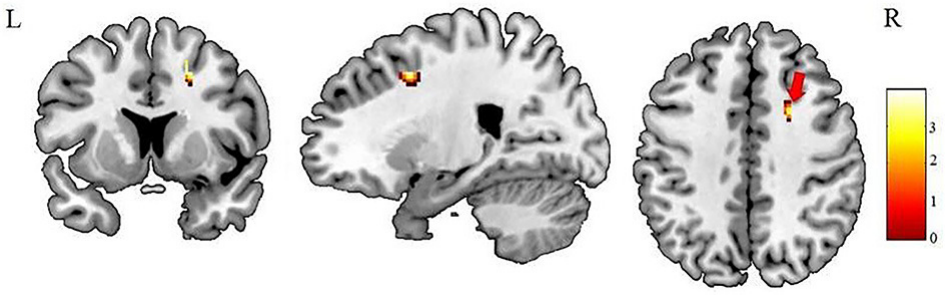
Patients with IGE showed increased FA (refer to color scale) in the deep WM of the right prefrontal lobe (as indicated by the red arrow) compared with healthy controls after AlphaSim correction (*P* < 0.05). FA, fractional anisotropy.

**Figure 3:**
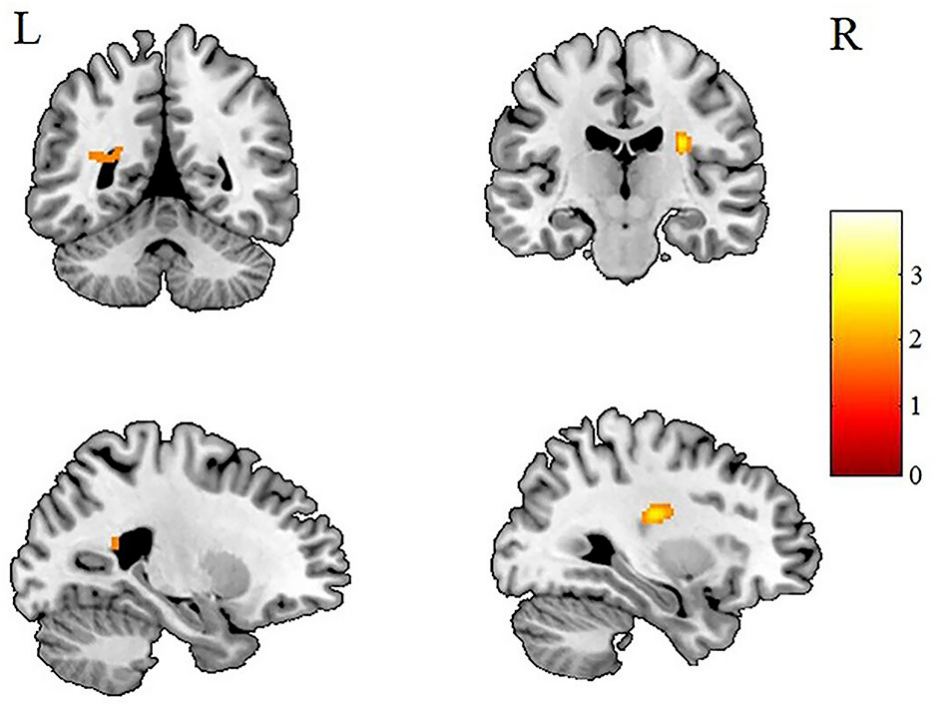
Patients with IGE showed increased AD (refer to color scale) in the WM of the left occipital lobe and the right corona radiata compared with healthy controls after AlphaSim correction (*P* < 0.05). AD, axial diffusivity; WM, white matter.

**Figure 4:**
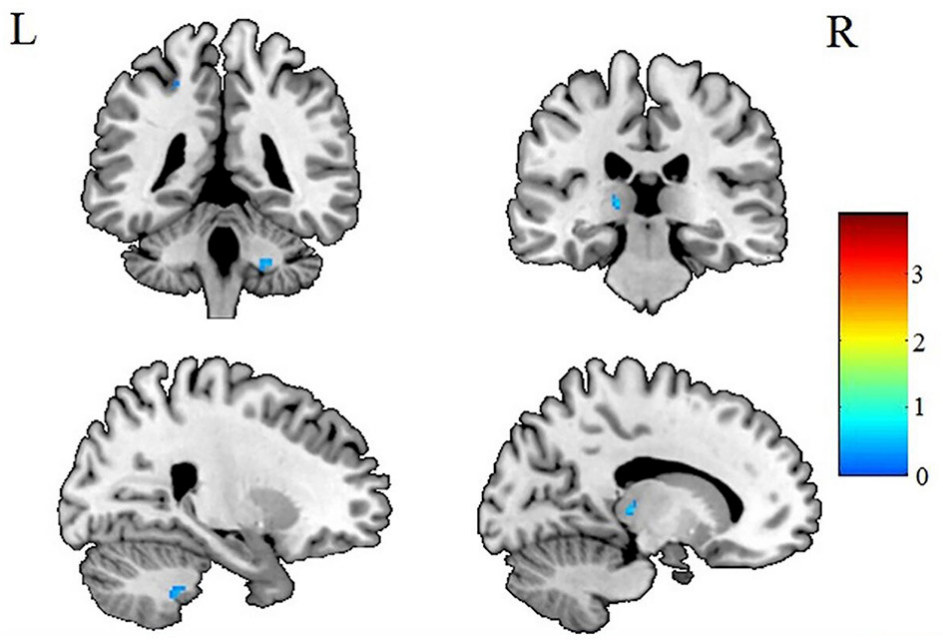
Patients with IGE showed decreased AD (refer to color scale) in the WM of the right middle cerebellar peduncle and left thalamus compared with healthy controls after AlphaSim correction (*P* < 0.05). AD, axial diffusivity; WM, white matter.

After AlphaSim correction, the patients with IGE showed significant alterations in several diffusion parameters compared with the HCs (Table 2). Compared with HCs, the IGE group had increased MD in the WM of the left splenium of the corpus callosum (Figure 1; Table 2), increased FA in the deep WM of the right frontal gyrus (Figure 2; Table 2), increased AD in the WM of the right corona radiata and left occipital lobe, and decreased AD in the WM of the left thalamus and right middle cerebellar peduncle (Figures 3, 4; Table 2). There were no significant correlations between the MD, FA, and AD values and any of the clinical measures.

**Table 2:** Location of brain regions with altered DTI measurements in patients with IGE.

**Cluster no**	**Cluster size**	***P*** **-value**	***t*** **-value**	**MNI coordinate**			**Region**
				**x**	**y**	**z**	
Increased FA	55	<0.001	3.96	22	10	44	Right frontal lobe
Increased MD	62	0.001	3.16	−26	−50	18	Center SCC
Increased AD	107	<0.001	3.96	28	−18	24	Center occipital lobe
	59	0.003	2.77	−34	−50	16	Right corona radiata
Decreased AD	43	<0.001	3.59	−18	−28	4	Center thalamus
	60	<0.001	3.42	22	−40	−42	Right middle cerebellar peduncle

*DTI, diffusion tensor imaging; IGE, idiopathic generalized epilepsy patients; FA, fractional anisotropy; MD, mean diffusivity; RD, radial diffusivity; AD, axial diffusivity; MNI, Montreal Neurological Institute; SCC, splenium of the corpus callosum*.

## Discussion

The current study investigated WM microstructural changes in children with new-onset, untreated IGE to help understand the dynamic microstructural alterations and the possible mechanisms leading to cognitive decline in the later, chronic stage of IGE. Consistent with previous studies (12, 19), we found increased MD in the left splenium of the corpus callosum (Figure 1). However, in contrast to the observations of decreased FA in WM of the frontal lobe in previous studies of chronic focal epilepsy and IGE (20–22), we observed increased FAs in the deep WM of the frontal lobe (Figure 2). In addition, we found both increased and decreased ADs in several brain areas (Figures 3, 4). None of these altered diffusion indices were associated with the clinical parameters. We will discuss these points in turn.

### Increased FA in the Deep WM of the Right Frontal Lobe

Our most striking observation is of increased FA in the deep WM of the right frontal lobe. The mesial frontal regions play a crucial role in the spreading and generalization of epileptic discharges in IGE (23, 24), and stimulation of the frontal cortex (25) elicits spike-wave phenomena suggesting that this area is essential for initiation or propagation of epileptiform activity. So abnormalities in this area are not unexpected and indeed have been observed: but what has been reported in both adults (20, 22) and children (21) with IGE is decreased FA in the prefrontal WM, the opposite of our finding. Also, our finding of increased FA in the deep WM of the right prefrontal lobe differ from the findings of previous studies in patients with new-onset epilepsy, one employing an ROI approach (12, 14) and the other using a whole-brain analysis (13). Such discrepancies between reported DTI findings might reflect technical factors, such as different sample sizes, antiepileptic medications, illness durations, and analytic methods. However, it is worth considering what maybe the implications for the underlying neuropathology.

Decreased FA has been interpreted as reflecting poor fiber coherence and myelination, often considered a hallmark of neurodegenerative and similar disorders (10). Increased FA has been reported more rarely, in neuropsychiatric diseases, such as Williams syndrome (26), bipolar disorder (27), multiple sclerosis (28), and preclinical Alzheimer's disease (29). The possible implications of higher FA include greater brain reserve, which might be complemented by or a manifestation of cognitive reserve (29), loss of dendrites and/or swelling of neuronal cell bodies (28), increases in myelination, microscopic deficits of axonal structures, or decreases in axonal diameter, packing density, and branching (9).

Another possible explanation of our finding of increased FA is a relationship to fiber crossings: several areas of the brain (e.g., the centrum semiovale, uncinate fasciculi, and transpontine fibers) with considerable fiber crossings have low FA (10, 30). It might be that the increased FA we observed in the deep WM of the right frontal lobe (which includes the centrum semiovale) might reflect a smaller number of WM crossings (more homogeneity in the underlying fiber orientation) than in controls.

Taking together the absence of FA abnormalities reported using a similar whole-brain analytic method in children with new-onset IGE, but with greater illness duration than in our study (13), and the decreased FA reported in chronic adult IGE (20, 22), we tentatively suggest that brain microstructural changes in IGE are dynamic, with increased FA in some brain areas in the early stages that progress to decreased FA in the chronic stage. The increased FA we observed may be particularly relevant early on, perhaps as part of a compensatory mechanism to maintain normal cognitive function, while the decreased FA reported in chronic epilepsy might reflect a cause of cognitive dysfunction. Future longitudinal imaging studies are needed to characterize changes over the course of IGE and their relationship with cognitive measurements.

### Lack of Correlation With Cognitive Deficit

Previous studies have reported a relationship between cognitive deficit and frontal dysfunction in IGE (15, 16), and between the duration of epilepsy and cognitive decline (15). A study (14) of new-onset JME revealed significantly worse results in neuropsychological tests and some correlations with FA values in some regions. Practical considerations did not allow us to perform more sophisticated tests than MMSE, for which we found no correlation with altered FA (or in fact any diffusion parameter). This may be because our patients, new-onset children with a limited duration of epilepsy, covered to narrow a range of structural and functional changes for such a correlation to reach statistical significance, at least for this relatively limited sample size. Alternatively, the balance of primary pathophysiology and compensatory changes early on may make the relationship function more complicated.

### Increased MD in the Left Splenium of the Corpus Callosum

Another notable finding of the current study is the increased MD in the left splenium of the corpus callosum in patients with IGE. Increased diffusivity is usually explained by excitotoxic mechanisms caused by seizures, which lead to cell lysis and death (31) and result in the expansion of the extracellular space. The corpus callosum plays a key role in connecting cognitive and sensory information between the bilateral hemispheres (32), and the posterior part of the corpus callosum connects the visual areas in the occipital lobe. Consistent with our result, increased MD in the splenium of the corpus callosum has been reported in children with idiopathic epilepsy (12) and patients with periventricular nodular heterotopia (33). The present study helps to demonstrate the important role of the corpus callosum in the early stage in children with IGE.

### Changes in AD

Changes in AD were quite complicated (Figures 3, 4). Although several areas showed abnormal AD, only one area exhibited abnormal FA or MD. Other studies have reported similar findings (13) of increased or decreased ADs in children with new-onset IGE accompanied by no abnormalities in FA, ADC, or RD. Why this should be so is not obvious. It may be relevant that a study (34) in the shiverer mouse model found that AD was the most sensitive marker of dysmyelination. The increased AD in the left occipital lobe may reflect an increased extra-axonal space in the occipital lobe. It may be relevant that the left splenium of the corpus callosum, which connects the visual areas in the occipital lobe, showed increased MD (Figure 2). Thus, we speculate that the occipital lobe sensitively reflects the early WM injury of epilepsy.

However, we did not find any alterations in RD, which is considered to reflect impaired myelin integrity or decreased axon fiber density in WM pathways (34). A study in the mouse (35) found that the absence of myelin increased RD with minimal influence on AD.

### Implications for Brain Circuitry

We found altered diffusion parameters in areas of the brain which have often been reported to be abnormal in epilepsy. In particular, the corona radiata, corpus callosum, thalamus, and frontal and occipital lobes frequently show abnormal diffusion indices in patients with IGE (14, 21, 22, 36). Abnormal thalamocortical circuitry plays an important role in the generation of PSW discharges (37, 38). The corona radiata and the frontal WM, where we found abnormalities, comprise the thalamofrontal connections, which make up the major epileptic brain network (38). The clinical efficacy of corpus callosotomy shows that the corpus callosum is the principal pathway of cross-hemisphere discharge synchronization and seizure spread (39). Our results are consistent with this thalamocortical circuit model in IGE. The varied abnormalities of diffusion metrics might suggest that seizure propagation is widespread in children with new-onset, untreated IGE.

## Limitations of the Study

First, the number of children studied was modest, and we were unable to conduct any subgroup analysis. Second, MMSE is not well-suited to evaluate subtle neuropsychological changes in new-onset epilepsy. Third, it is of course difficult to fully interpret the DTI findings in the absence of histopathological assessments: future studies using animal models of epilepsy may help with this. Finally, this cross-sectional study cannot address the dynamic evolution of these abnormalities: longitudinal follow-ups will help to elucidate this.

In summary, our results indicate that microstructural abnormalities exist from the very beginning of IGE and that AD may be more affected than FA, MD, and RD in the initial stages in children with IGE. Calculating all the diffusion parameters allows the detection of more WM microstructural changes in children with IGE than simply analyzing FA and MD. The increased FA in children with new-onset, untreated IGE differs markedly from chronic patients with IGE, perhaps evidence of gradual progression of cognitive impairments and possible compensatory mechanisms in IGE prior to cognitive decline. Longitudinal studies are needed to determine how the FA and other diffusion parameters change over the course of IGE and the potential impact of therapeutic intervention on these parameters.

## Data Availability Statement

The original contributions presented in the study are included in the article/supplementary material, further inquiries can be directed to the corresponding author/s.

## Ethics Statement

The studies involving human participants were reviewed and approved by Ethics Committee of Yibin Second People's Hospital. Written informed consent to participate in this study was provided by the participants' legal guardian/next of kin.

## Author Contributions

All authors listed have made a substantial, direct, and intellectual contribution to the work and approved it for publication.

## Funding

This study was supported by the funding of Changjiang Scholar Professorship Award (award no. T2014190), CMB Distinguished Professorship Award (award no. F510000/G16916411), Changjiang Scholars and Innovative Research Team in the University of China (PCSIRT, grant no. IRT16R52), the National Natural Science Foundation (grant no. 81621003), the Natural Science Youth fund of Southwest Medical University (grant no. 2017-ZRQN-086), Project of Sichuan Health Committee (grant no. 20PJ309), and the Key Project of Yibin Science and Technology Bureau of Sichuan Province (2013SF009).

## Conflict of Interest

The authors declare that the research was conducted in the absence of any commercial or financial relationships that could be construed as a potential conflict of interest.

## Publisher's Note

All claims expressed in this article are solely those of the authors and do not necessarily represent those of their affiliated organizations, or those of the publisher, the editors and the reviewers. Any product that may be evaluated in this article, or claim that may be made by its manufacturer, is not guaranteed or endorsed by the publisher.

## References

[1] Ngugi AK Bottomley C Kleinschmidt I Sander JW Newton C. Estimation of the burden of active and life-time epilepsy: a meta-analytic approach. Epilepsia. (2010) 51:883–90. 10.1111/j.1528-1167.2009.02481.x 20067507 PMC3410521

[2] Neufeld MY Vishne T Chistik V Korczyn AD. Life-long history of injuries related to seizures. Epilepsy Res. (1999) 34:123–7. 10.1016/S0920-1211(98)00105-3 10210026

[3] Marini C King MA Archer JS Newton MR Berkovic SF. Idiopathic generalised epilepsy of adult onset: clinical syndromes and genetics. J Neurol Neurosurg Psychiatry. (2003) 74:192–6. 10.1136/jnnp.74.2.192 12531947 PMC1738270

[4] Jallon P Latour P. Epidemiology of idiopathic generalized epilepsies. Epilepsia. (2005) 9(Suppl. 46):10–4. 10.1111/j.1528-1167.2005.00309.x 16302871

[5] Fisher RS Cross JH D'Souza C French JA Haut SR Higurashi N. Instruction manual for the ILAE 2017 operational classification of seizure types. Epilepsia. (2017) 58:531–42. 10.1111/epi.13671 28276064

[6] Scheffer IE Berkovic S Capovilla G Connolly MB French J Guilhoto L. ILAE classification of the epilepsies: position paper of the ILAE commission for classification and terminology. Epilepsia. (2017) 58:512–21. 10.1111/epi.13709 28276062 PMC5386840

[7] Basser PJ Pierpaoli C. Microstructural and physiological features of tissues elucidated by quantitative-diffusion-tensor MRI. J Magn Reson B. (1996) 111:209–19. 10.1006/jmrb.1996.0086 8661285

[8] Klingberg T Hedehus M Temple E Salz T Gabrieli JD Moseley ME. Microstructure of temporo-parietal white matter as a basis for reading ability: evidence from diffusion tensor magnetic resonance imaging. Neuron. (2000) 25:493–500. 10.1016/S0896-6273(00)80911-3 10719902

[9] Beaulieu C. The basis of anisotropic water diffusion in the nervous system - a technical review. NMR Biomed. (2002) 15:435–55. 10.1002/nbm.782 12489094

[10] Alexander AL Lee JE Lazar M Field AS. Diffusion tensor imaging of the brain. Neurotherapeutics. (2007) 4:316–29. 10.1016/j.nurt.2007.05.011 17599699 PMC2041910

[11] Carlson HL Laliberte C Brooks BL Hodge J Kirton A Bello-Espinosa L. Reliability and variability of diffusion tensor imaging (DTI) tractography in pediatric epilepsy. Epilepsy Behav. (2014) 37:116–22. 10.1016/j.yebeh.2014.06.020 25014749

[12] Hutchinson E Pulsipher D Dabbs KA y Gutierrez AM Sheth R Jones J. Children with new-onset epilepsy exhibit diffusion abnormalities in cerebral white matter in the absence of volumetric differences. Epilepsy Res. (2010) 88:208–14. 10.1016/j.eplepsyres.2009.11.011 20044239 PMC2826144

[13] Widjaja E Kis A Go C Raybaud C Snead OC Smith ML. Abnormal white matter on diffusion tensor imaging in children with new-onset seizures. Epilepsy Res. (2013) 104:105–11. 10.1016/j.eplepsyres.2012.10.007 23182414

[14] Ekmekci B Bulut HT Gumustas F Yildirim A Kustepe A. The relationship between white matter abnormalities and cognitive functions in new-onset juvenile myoclonic epilepsy. Epilepsy Behav. (2016) 62:166–70. 10.1016/j.yebeh.2016.07.015 27484748

[15] Pascalicchio TF de Araujo Filho GM da Silva Noffs MH Lin K Caboclo LO Vidal-Dourado M. Neuropsychological profile of patients with juvenile myoclonic epilepsy: a controlled study of 50 patients. Epilepsy Behav. (2007) 10:263–7. 10.1016/j.yebeh.2006.11.012 17258506

[16] Piazzini A Turner K Vignoli A Canger R Canevini M P. Frontal cognitive dysfunction in juvenile myoclonic epilepsy. Epilepsia. (2008) 49:657–62. 10.1111/j.1528-1167.2007.01482.x 18177360

[17] Zhang T Chen L Wang Y Zhang M Wang L Xu X. Impaired theory of mind in Chinese children and adolescents with idiopathic generalized epilepsy: association with behavioral manifestations of executive dysfunction. Epilepsy Behav. (2018) 79:205–12. 10.1016/j.yebeh.2017.12.006 29309954

[18] Epilepsy A. Proposal for revised classification of epilepsies and epileptic syndromes. Epilepsia. (1989) 30:389–99. 10.1111/j.1528-1157.1989.tb05316.x 2502382

[19] Lyra KP Chaim KT Leite CC Park EJ Andrade CS Passarelli V. Corpus callosum diffusion abnormalities in refractory epilepsy associated with hip pocampal sclerosis. Epilepsy Res. (2017) 137:112–8. 10.1016/j.eplepsyres.2017.09.008 28988018

[20] Deppe M Kellinghaus C Duning T Moddel G Mohammadi S Deppe K. Nerve fiber impairment of anterior thalamocortical circuitry in juvenile myoclonic epilepsy. Neurology. (2008) 71:1981–5. 10.1212/01.wnl.0000336969.98241.17 19064879

[21] Yang T Guo Z Luo C Li Q Yan B Liu L. White matter impairment in the basal ganglia-thalamocortical circuit of drug-naive childhood absence epilepsy. Epilepsy Res. (2012) 99:267–73. 10.1016/j.eplepsyres.2011.12.006 22227509

[22] Focke NK Diederich C Helms G Nitsche MA Lerche H Paulus W. Idiopathic-generalized epilepsy shows profound white matter diffusion-tensor imaging alterations. Hum Brain Mapp. (2014) 35:3332–42. 10.1002/hbm.22405 25050427 PMC6869818

[23] Isnard J Guenot M Sindou M Mauguiere F. Clinical manifestations of insular lobe seizures: a stereo-electroencephalographic study. Epilepsia. (2004) 45:1079–90. 10.1111/j.0013-9580.2004.68903.x 15329073

[24] Gotman J Grova C Bagshaw A Kobayashi E Aghakhani Y Dubeau F. Generalized epileptic discharges show thalamocortical activation and suspension of the default state of the brain. Proc Natl Acad Sci USA. (2005) 102:15236–40. 10.1073/pnas.0504935102 16217042 PMC1257704

[25] Bancaud J Talairach J Morel P Bresson M Bonis A Geier S. “Generalized” epileptic seizures elicited by electrical stimulation of the frontal lobe in man. Electroencephalogr Clin Neurophysiol. (1974) 37:275–82. 10.1016/0013-4694(74)90031-5 4136279

[26] Hoeft F Barnea-Goraly N Haas BW Golarai G Ng D Mills D. More is not always better: increased fractional anisotropy of superior longitudinal fasciculus associated with poor visuospatial abilities in williams syndrome. J Neurosci. (2007) 27:11960–5. 10.1523/JNEUROSCI.3591-07.2007 17978036 PMC6673356

[27] Yurgelun-Todd DA Silveri MM Gruber SA Rohan M Rohan ML Pimentel PJ. White matter abnormalities observed in bipolar disorder: a diffusion tensor imaging study. Bipolar Disord. (2007) 9:504–12. 10.1111/j.1399-5618.2007.00395.x 17680921

[28] Hannoun S Durand-Dubief F Confavreux C Ibarrola D Streichenberger N Cotton F. Diffusion tensor-MRI evidence for extra-axonal neuronal degeneration in caudate and thalamic nuclei of patients with multiple sclerosis. AJNR Am J Neuroradiol. (2012) 33:1363–8. 10.3174/ajnr.A2983 22383236 PMC7965527

[29] Racine AM Adluru N Alexander AL Christian BT Okonkwo OC Oh J. Associations between white matter microstructure and amyloid burden in preclinical Alzheimer's disease: a multimodal imaging investigation. Neuroimage Clin. (2014) 4:604–14. 10.1016/j.nicl.2014.02.001 24936411 PMC4053642

[30] Alexander AL Hurley SA Samsonov AA Adluru N Hosseinbor AP Mossahebi P. Characterization of cerebral white matter properties using quantitative magnetic resonance imaging stains. Brain Connect. (2011) 1:423–46. 10.1089/brain.2011.0071 22432902 PMC3360545

[31] Yogarajah M Duncan JS. Diffusion-based magnetic resonance imaging and tractography in epilepsy. Epilepsia. (2008) 49:189–200. 10.1111/j.1528-1167.2007.01378.x 17941849

[32] van der Knaap LJ van der Ham IJ. How does the corpus callosum mediate interhemispheric transfer? A review. Behav Brain Res. (2011) 223:211–21. 10.1016/j.bbr.2011.04.018 21530590

[33] Liu W An D Niu R Gong Q Zhou D. Integrity of the corpus callosum in patients with periventricular nodular heterotopia related epilepsy by FLNA mutation. Neuroimage Clin. (2018) 17:109–14. 10.1016/j.nicl.2017.10.002 29062687 PMC5647519

[34] Tyszka JM Readhead C Bearer EL Pautler RG Jacobs RE. Statistical diffusion tensor histology reveals regional dysmyelination effects in the shiverer mouse mutant. Neuroimage. (2006) 29:1058–65. 10.1016/j.neuroimage.2005.08.037 16213163 PMC3376084

[35] Song SK Sun SW Ramsbottom MJ Chang C Russell J Cross AH. Dysmyelination revealed through MRI as increased radial (but unchanged axial) diffusion of water. Neuroimage. (2002) 17:1429–36. 10.1006/nimg.2002.1267 12414282

[36] Groppa S Moeller F Siebner H Wolff S Riedel C Deuschl G. White matter microstructural changes of thalamocortical networks in photosensitivity and idiopathic generalized epilepsy. Epilepsia. (2012) 53:668–76. 10.1111/j.1528-1167.2012.03414.x 22360784

[37] Danober L Deransart C Depaulis A Vergnes M Marescaux C. Pathophysiological mechanisms of genetic absence epilepsy in the rat. Prog Neurobiol. (1998) 55:27–57. 10.1016/S0301-0082(97)00091-9 9602499

[38] Blumenfeld H. Cellular and network mechanisms of spike-wave seizures. Epilepsia. (2005) 9(Suppl. 46):21–33. 10.1111/j.1528-1167.2005.00311.x 16302873

[39] Matsuo A Ono T Baba H Ono K. Callosal role in generation of epileptiform discharges: quantitative analysis of EEGs recorded in patients undergoing corpus callosotomy. Clin Neurophysiol. (2003) 114:2165–71. 10.1016/S1388-2457(03)00234-7 14580615

